# Enrichment analysis for spatial and single-cell metabolomics accounting for molecular ambiguity

**DOI:** 10.1093/bioadv/vbaf100

**Published:** 2025-05-21

**Authors:** Bishoy Wadie, Martijn R Molenaar, Lucas M Vieira, Theodore Alexandrov

**Affiliations:** Structural and Computational Biology Unit, European Molecular Biology Laboratory (EMBL), Heidelberg 69117, Germany; Faculty of Biosciences, Collaboration for Joint PhD Degree between EMBL and Heidelberg University, Heidelberg 69047, Germany; Structural and Computational Biology Unit, European Molecular Biology Laboratory (EMBL), Heidelberg 69117, Germany; Structural and Computational Biology Unit, European Molecular Biology Laboratory (EMBL), Heidelberg 69117, Germany; Department of Pharmacology, University of California San Diego, San Diego, CA 92037, United States; Structural and Computational Biology Unit, European Molecular Biology Laboratory (EMBL), Heidelberg 69117, Germany; Department of Pharmacology, University of California San Diego, San Diego, CA 92037, United States; Department of Bioengineering, University of California San Diego, San Diego, CA 92037, United States; DeepCyte Inc, San Diego, CA 92122, United States

## Abstract

**Summary:**

Imaging mass spectrometry (imaging MS) has advanced spatial and single-cell metabolomics, but the reliance on MS1 data complicates the accurate identification of molecular structures, not being able to resolve isomeric and isobar molecules. This prevents application of conventional methods for overrepresentation analysis (ORA) and metabolite set enrichment analysis (MSEA). To address this, we introduce *S2IsoMEr* R package and a web app for METASPACE, which uses bootstrapping to propagate isomeric/isobaric ambiguities into the enrichment analysis. We demonstrate *S2IsoMEr* for single-cell metabolomics and the METASPACE web app for spatial metabolomics.

**Availability and implementation:**

METASPACE web app can be used on existing and new datasets submitted to METASPACE (https://metaspace2020.org). The source code for the *S2IsoMEr* R package is available on GitHub (https://github.com/alexandrovteam/S2IsoMEr).

## 1 Introduction

Recent advances in imaging mass spectrometry (imaging MS) have boosted the emerging fields of spatial metabolomics and lipidomics as well as opened novel avenues for obtaining metabolomics data on the single-cell level. However, imaging MS data are usually collected in the MS1 mode, without the untargeted MS/MS tandem fragmentation commonly used in bulk metabolomics. Molecular annotation of MS1 data provides identities on the Level 2 (putatively annotated compounds) of the Metabolomics Standards Initiative (Sumner *et al.* 2007), mainly relying on the m/z values of ions and their isotopic peaks (Palmer *et al.* 2017). As a result, molecular candidates with identical (‘isomers’) or similar (‘isobars’) mass-to-charge (m/z) values cannot be resolved.

This unavoidable ambiguity in the identification of metabolites complicates downstream analyses such as overrepresentation analysis (ORA) and metabolite set enrichment analysis (MSEA) (Xia and Wishart 2010). A common way to handle the ambiguous identification in imaging MS is to manually curate the candidates and select the most plausible ones. However, this does not always remove the molecular ambiguity, introduces a selection bias and is time-consuming, especially when the number of metabolites is large.

Here, we present two implementations of a new method to perform metabolite enrichment analysis that addresses the challenge of metabolite identification ambiguity. The first is a web app on METASPACE (Palmer *et al.* 2017). The second, S2IsoMEr, is a new R package designed for (spatial) single-cell metabolomics datasets. In both implementations, the key idea to handle molecular isomers and/or isobars is to propagate the molecular ambiguity into the analysis by iterative random sampling (hereafter, bootstrapping) of isomeric/isobaric candidates.

## 2 Description

METASPACE is an online platform for metabolite identification in spatial metabolomics allowing users to upload imaging MS datasets and to perform annotation of metabolites and lipids in a false discovery rate (FDR)-controlled manner (Palmer *et al.* 2017). In the annotation process, METASPACE makes use of databases containing molecular formulas of biological interest. For each molecular formula, METASPACE calculates a score, based on which a corresponding FDR value is assigned. Because METASPACE performs annotation based on the MS1 data, it reports multiple possible isomers and isobars for the annotated ions which is mainly determined by the chosen annotation database (e.g. HMDB). As a result, each annotated ion may be associated with a large number of molecular structures.

**Figure 1.**
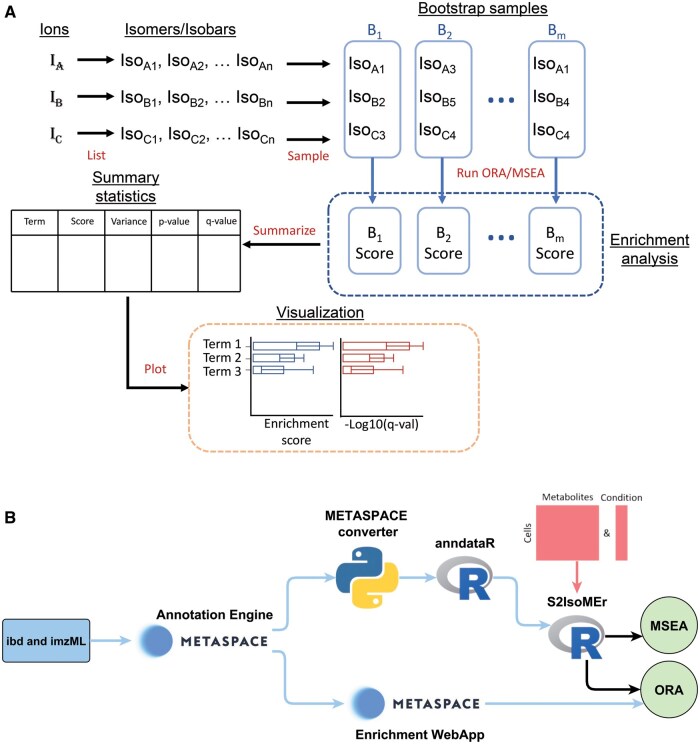
Overview of the bootstrapping-based enrichment and implementations. (A) Illustration of molecular ambiguity handling with bootstrapping and result reporting. All references to scores indicate enrichment scores. (B) Flowchart of input for the enrichment web app and *S2IsoMEr*, supporting both spatial and single-cell metabolomics workflows.

Due to this annotation ambiguity, performing enrichment analysis of molecule names using a classical approach is not possible. Accordingly, we leveraged bootstrapping to consider such ambiguity in the enrichment analysis. In each iteration, one molecular candidate for each ion is randomly sampled (with replacement) from all isomeric and/or isobaric candidates corresponding to that ion (Fig. 1A), optionally using weights indicating their likelihood or relative abundance. By default, all isomers and isobars are assigned equal weights. However, users have the flexibility to adjust these weights based on various criteria. For example, isomers that are more frequently detected can be given higher weights, those referenced in biological pathways can be prioritized, or experimental data (e.g. MS/MS) can be used to downweight isomers that are unlikely to be detected. Regarding bootstrapping iterations, the number of iterations is chosen to balance computational efficiency and statistical robustness. It depends on the level of ambiguity in the input annotations, with higher ambiguity requiring more iterations for more reliable results. While having more iterations generally improves stability, we recommend a minimum of 100 bootstraps to ensure robust results while keeping computation time manageable. After running enrichment for each bootstrap, aggregate statistics of the individual enrichment analyses are calculated and reported for each term, highlighting the variation across bootstraps (Fig. 1A). More details on the methods are available in Supplementary Notes S1.1 and S1.2, while information on computational performance with different dataset sizes and bootstrapping iterations can be found in Supplementary Note S5 and Supplementary Fig. S7.

Regarding the property databases (hereafter metabolite sets) required for enrichment analyses, the algorithm supports LION ontology for lipids (Molenaar *et al.* 2019) and multiple class-based and pathway-based sets for metabolites and lipids curated from RAMP-DB (Braisted *et al.* 2023) (Supplementary Note S2). This database encompasses biological pathways and metabolic classes, which are further categorized into super, main, and sub-classes. The pathways from RAMP-DB integrate multiple resources, including SMPDB, Reactome, KEGG, and WikiPathways (Kanehisa and Goto 2000, Frolkis *et al.* 2010, Kutmon *et al.* 2016, Fabregat *et al.* 2018).

In the METASPACE web app implementation for ORA, the user selects a metabolite set when submitting a dataset. Once processing has finished, users can access the enrichment web app and view the results. Bootstrapping is performed as mentioned previously on the chosen set and enrichment of each term is assessed against its presence in the full molecular database in each bootstrap sample using a one-tailed Fisher’s exact test. The resulting *P*-values from the Fisher’s exact test are adjusted for multiple testing using the Benjamini and Hochberg method (Benjamini and Hochberg 1995) and median intersection size along with fold enrichment score (Wu *et al.* 2021) are reported per term (Supplementary Note S1.4). Bar plots present the aggregated statistics from the hypergeometric tests across bootstraps, and users can adjust significance thresholds, FDR cutoffs, and explore the enriched annotations for each term (Supplementary Fig. S1).

Although imaging MS is primarily known for spatial metabolomics, it has also been increasingly adopted for single-cell metabolomics as well (Rappez *et al.* 2021, Capolupo *et al.* 2022). The resulting datasets are matrices consisting of *n* metabolites measured for *m* single cells. While the METASPACE implementation performs ORA of metabolite annotations from one dataset (as compared to all metabolites from the database used for annotation), *S2IsoMEr* is currently designed for single-cell metabolomics and supports both ORA and MSEA (Fig. 1B). It makes use of the same metabolite sets available in the METASPACE web app and can be run with or without accounting for isomeric/isobaric ambiguity. The decision tree in Supplementary Fig. S2 provides guidance on how to select the appropriate enrichment type. Moreover, *S2IsoMEr* can be extended to support spatial metabolomics datasets from METASPACE using *metaspace-converter* (https://github.com/metaspace2020/metaspace-converter). This tool converts metaspace datasets to an anndata object and can subsequently be loaded into R with the *anndataR* R package (Virshup *et al.* 2021, 2023) to provide single-pixel matrices for *S2IsoMEr* (Fig. 1B). *S2IsoMEr* requires as inputs (i) a matrix containing the single-cell metabolomics measurements, (ii) a vector indicating the condition or group to which each cell belongs, and (iii) the conditions of interest to compare (e.g. for comparing two conditions or cell types). It is assumed that cells from both groups share the same metabolite annotations but differ in their intensities. Therefore, the user has the option to perform MSEA by ranking all metabolites prior to enrichment. More information on ranking metrics are available in Supplementary Note S1.3.

## 3 Case studies

### 3.1 Bootstrapping-based ORA of spatial metabolomics dataset in METASPACE

We showcase ORA in METASPACE using a mouse brain dataset (https://metaspace2020.eu/dataset/2022-05-31_10h46m34s). For the enrichment analysis, we selected the CoreMetabolome database (Wadie *et al.* 2024) as an annotation database with an FDR cutoff of 10%, excluded off-sample annotations as implemented in METASPACE and performed ORA using LION ontology as metabolite set (settings: Database=CoreMetabolome, molecule type=lipid, category=class, ontology=LION). More details on settings and visualization of enrichment results in webapp are available in Supplementary Note S3.

As anticipated, LION terms associated with sphingolipids such as ‘phosphosphingolipids’ and ‘sphingolipids’ were found to be highly overrepresented (Supplementary Fig. S1) consistent with previous reports that brain tissues have relatively high amounts of sphingolipids (Olsen and Færgeman 2017, Hussain *et al.* 2019).

### 3.2 MSEA of single-cell metabolomics data with the S2IsoMEr R package

To showcase *S2IsoMEr*, we applied it to our previously reported single-cell metabolomics dataset (Rappez *et al.* 2021) of HepaRG cells, which models NASH by stimulating HepaRG cells with fatty acids and other inhibitors compared to a healthy control, followed by MALDI imaging MS. In total, there were 3 perturbation conditions and 1 control condition, each with 4 replicates, resulting in 16 datasets in METASPACE (Supplementary Table S1). Detailed methods on preparation of single-cell data as input for S2IsoMEr are available in Supplementary Note S4.

We compared HepaRG cells treated with exogenous fatty acids (steatosis model, ‘F’) to untreated cells (healthy state, ‘U’). A bootstrapping metabolite enrichment analysis with 100 iterations was performed, accounting for isomeric and isobaric structures. As anticipated and consistent with previous analyses, terms associated with fat accumulation, such as ‘triacylglycerols’ and ‘diradylglycerols’, were highly enriched in steatotic cells, while ‘glycerophosphocholines’ and ‘glycerophosphoethanolamines’ were enriched in healthy cells, in both ORA and MSEA (Supplementary Figs S4 and S5). The findings were also consistent when the other metabolic states were compared to healthy cells (Supplementary Fig. S6).

### 3.3 Comparison between bootstrapping-based and traditional ORA

To highlight the added value of bootstrapping, we applied both standard and bootstrapped ORA using *S2IsoMEr* on the same dataset as the previous case study. The standard ORA was performed against molecular formulas, ignoring isomeric/isobaric ambiguity.

Considering only terms significantly enriched in standard ORA, ‘Glycerophosphoethanolamines’, ‘Fatty acyls’, and ‘Linoleic acids and derivatives’ were not enriched in the bootstrapping-based approach, which is likely explained by their low (<0.5) ambiguity score (Supplementary Fig. S8). This illustrates how the bootstrapping approach helps identify false positives in standard ORA by highlighting terms that are prone to high ambiguity in the input annotations. More information on ambiguity score is available in Supplementary Note S1.5.

## 4 Conclusion

Ambiguous identification of metabolites and lipids in spatial and single-cell metabolomics by imaging MS complicates downstream enrichment analysis approaches that are routinely performed on bulk metabolomics data. In this work, we described a method of bootstrapping enrichment analysis to address this problem by performing the analysis multiple times, with iterative random sampling of the molecular candidates belonging to each ion. The implementation of bootstrapping-based enrichment in METASPACE provides an accessible way to quickly explore the coverage of lipid/metabolite classes and properties in imaging MS datasets submitted to METASPACE. In addition, the *S2IsoMEr* R package extends the bootstrapping approach to ranking-based enrichment in single-cell metabolomics datasets and we foresee a wide adoption of the package as more single-cell datasets are generated.

## Author contributions

Bishoy Wadie (Conceptualization, Data curation, Formal analysis, Writing – original draft, Writing – review & editing, Visualization, Software), Martijn R. Molenaar (Conceptualization, Data curation, Formal analysis, Writing – original draft, Writing – review & editing, Software), Lucas M. Vieira (Data curation, Writing – original draft, Writing – review & editing, Visualization, Software), Theodore Alexandrov (Writing – review & editing, Supervision, Project administration, Funding acquisition). All authors have read and agreed to the published version of the manuscript.

## Supplementary Material

vbaf100_Supplementary_Data

## Data Availability

The single-cell metabolomics dataset used to showcase *S2IsoMEr is* available in *MetaboLights* at https://www.ebi.ac.uk/metabolights/MTBLS78 and can be accessed with *accession number MTBLS78*.
