# Supplementary material for: Enrichment analysis for spatial and single-cell metabolomics accounting for molecular ambiguity
Source: Bioinform Adv. 2025 May 21;5(1):vbaf100. doi: 10.1093/bioadv/vbaf100 (PMC12158160; doi:10.1093/bioadv/vbaf100)
Supplement: vbaf100_Supplementary_Data [file vbaf100_supplementary_data.zip › Bioinformatics_advances_Supplementary information.docx]

Supplementary information

**Enrichment analysis for spatial and single-cell metabolomics accounting for molecular ambiguity**

*Bishoy Wadie^1,2,*^, Martijn R. Molenaar^1,*^, Lucas Maciel Vieira^1,3^, Theodore Alexandrov^1,3,4,5,+^*

*^1^ Structural and Computational Biology Unit, European Molecular Biology Laboratory (EMBL), Heidelberg, Germany; ^2^ Collaboration for joint PhD degree between EMBL and Heidelberg University, Faculty of Biosciences, Heidelberg, Germany; ^3^ Department of Pharmacology, University of California San Diego, La Jolla, CA, USA; ^4^ Department of Bioengineering, University of California San Diego, La Jolla, CA, USA; ^5^ DeepCyte Inc., San Diego, CA, USA;*

*^*^ Equal contribution; ^+^ Corresponding author*

##

## **Supplementary Note 1:**

### Bootstrapping

To generate a bootstrapped sample from a list of possible isomeric/isobaric molecules, we employed a resampling strategy. For each of the *N* bootstrapping iterations, the process involves sampling 1 molecule from the list of isomers/isobars for each given ion in the annotation list. It is assumed that all isomers/isobars are equally likely and have the same sampling probability. However, annotation weights can be provided in *S2IsoMEr* *a-priori*.

Given:

- *M* : Number of input ions
- *Iso_i_* : Set of isomers/isobars (molecules) associated with ion *i*, where
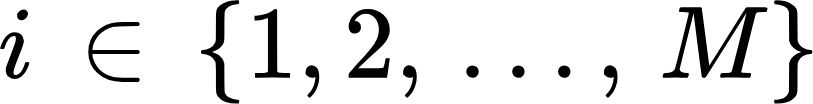
 and the size of *Iso_i_* may vary for each ion.
- *w_i_ :* The vector of sampling probabilities (weights) corresponding to the isomers in *Iso*_i_

For each ion
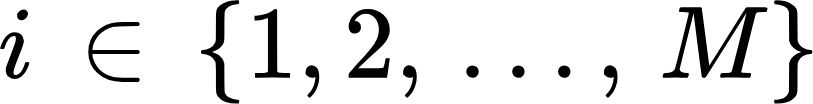
, a single molecule *x_i_* is sampled from *Iso_i_* . This process is repeated for *N* bootstrapping iterations.

The bootstrapping process can be expressed as:


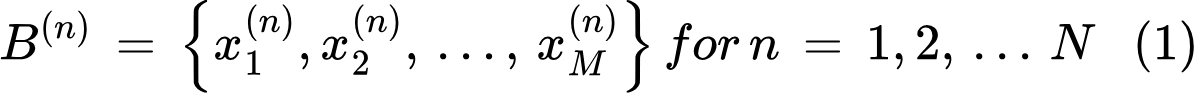


Where:

-
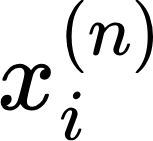
 is the isomer sampled from *Iso*_i_ during the *n-th* iteration.
-
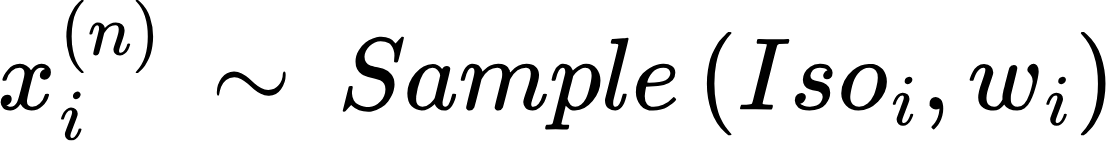
, where “Sample” represents a weighted random selection using *w_i_* as the probabilities.

### ORA contingency table adjustment

Overrepresentation analysis (ORA) in *S2IsoMEr* is performed using re-implementation of the “run_ora” function from the decoupleR package [(Badia-I-Mompel et al. 2022)](https://paperpile.com/c/5gCIgH/BoLmi). Everything is performed as standard ORA with a one-tailed fisher exact test. Additionally, since each observed ion typically corresponds to only a single isomer in the bootstrapping process, whereas multiple isomers of the same molecular formula may exist in the expected molecules for a given term in the chosen metabolite set, special handling was applied. Specifically, false negatives were filtered to exclude the remaining isomers for a given formula if the sampled isomer is a true positive (i.e. mapped to the given term in the metabolite set) to avoid inflating the count of false negatives due to redundant representations of the same molecule.

### MSEA signed ranking

In MSEA, metabolites are ranked based on their intensity changes between the conditions under comparison. In *S2IsoMEr* we implemented 4 ranking metrics : Log Fold Change (LFC), Wilcoxon rank-sum statistic, t-test statistic and BWS [(Zyla et al. 2017)](https://paperpile.com/c/5gCIgH/qWz7W). LFC measures the ratio of intensity levels between two conditions on a logarithmic scale, indicating how much a metabolite abundance increases or decreases. Positive LFC values show higher abundance in the test condition, while negative values indicate lower abundance. The Wilcoxon rank-sum is a non-parametric method that ranks all metabolites from both conditions and compares the sum of ranks to assess differences without assuming normal distribution. On the other hand, t-test is a parametric test that compares the means of two conditions under the assumption of normality. We use the statistic value exclusively for running MSEA, and to improve result interpretation, we multiply these statistical values by the sign of the Log Fold Change (LFC). This approach ensures that the direction of regulation (up or down) is incorporated while maintaining the magnitude of the statistic.

1. LFC :


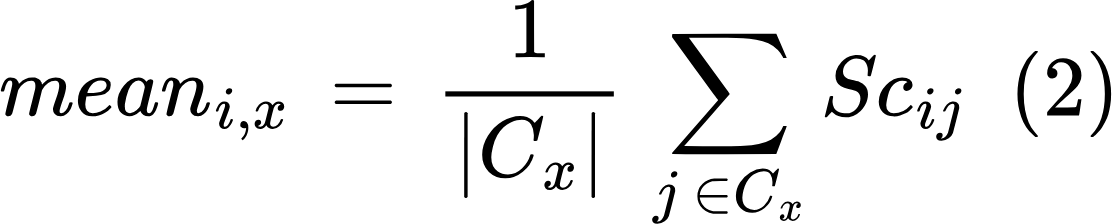


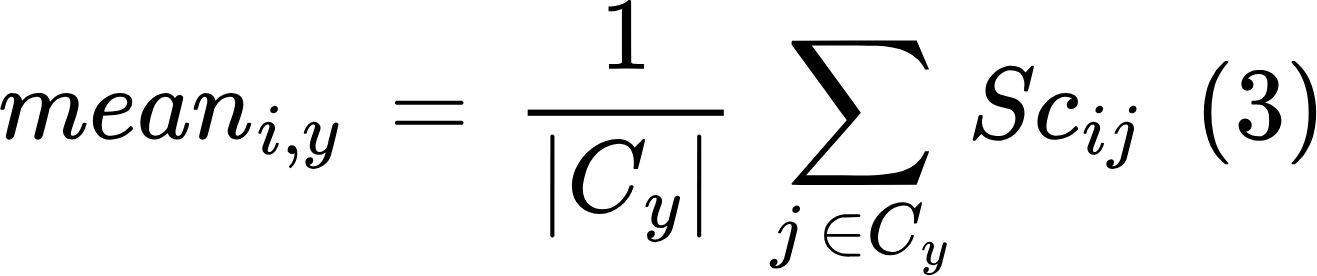


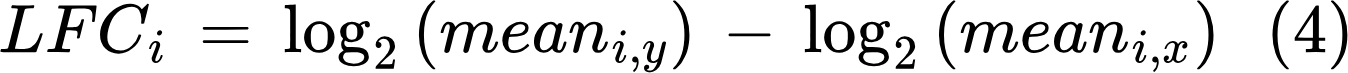


Where
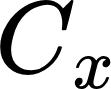
 and
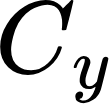
 are the sets of cell indices for the reference and test conditions, respectively.
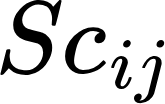
 represent the log10 intensity level of metabolite
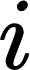
 in cell
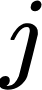
 .
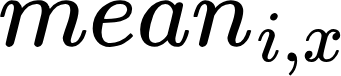
 and
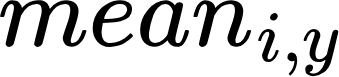
 are the mean of log10 intensity of metabolite
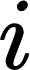
 in reference and test conditions, respectively.

1. Wilcoxon rank-sum statistic


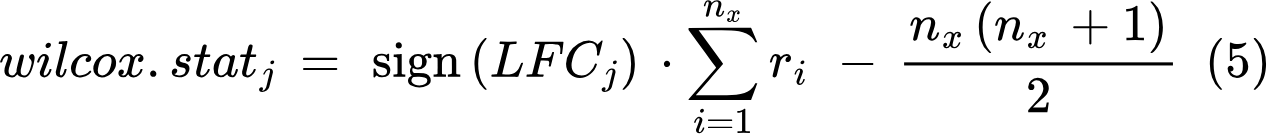


Where
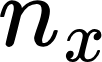
 is the number of cells in test condition *x,
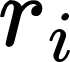
* denotes the rank of the *i-th* cell in both reference and test conditions for metabolite
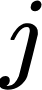
, and
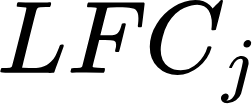
 is LFC of metabolite
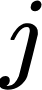
.

1. BWS


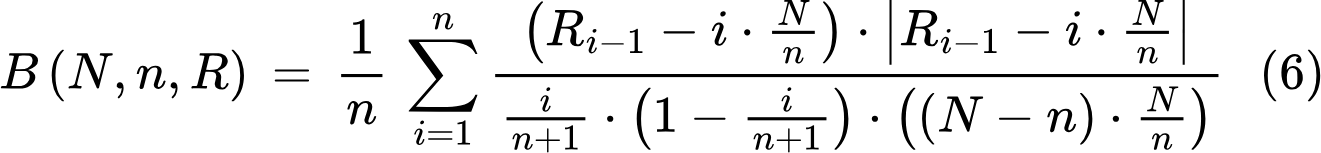


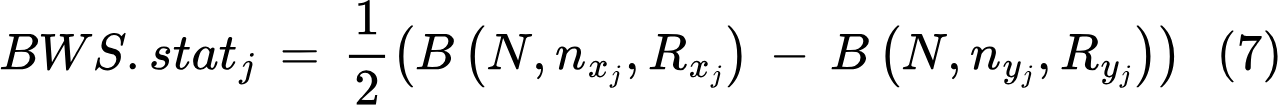


The above equation represents the modified one-tailed (greater) Neuhauser's difference statistic denoted by Murkami as a modification of the Baumgartner-Weiss-Schindle (BWS) statistic [(Murakami 2012)](https://paperpile.com/c/5gCIgH/fJ8jX).

-
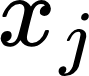
 : Intensity of metabolite
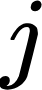
 in cells of test condition
-
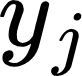
 : Intensity of metabolite
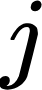
 in cells of Reference condition
-
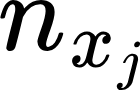
 : Number of cells in test condition
-
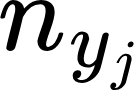
 : Number of cells in Reference condition
-
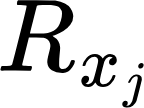
 : Rank of
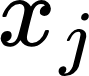
 relative to
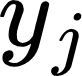

-
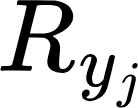
 : Rank of
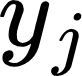
 relative to
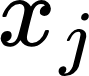

-
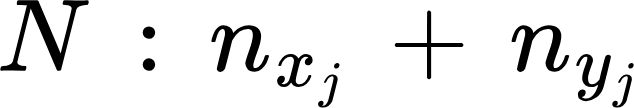


### Enrichment scores

In MSEA, the reported enrichment score is the Normalized Enrichment Score (NES) in the fgsea (fast gene set enrichment analysis) method [(Korotkevich et al. 2021)](https://paperpile.com/c/5gCIgH/AXznV). NES is a metric that adjusts the raw enrichment score (ES) to account for differences in metabolite set sizes. In MSEA, the enrichment score indicates how well a set of metabolites is represented at the extremes (top or bottom) of a ranked list of metabolites. The NES is calculated by normalizing the ES to a distribution of scores obtained from random permutations of the metabolite sets. For ORA, we use the fold enrichment (FE) score which measures how much a specific metabolite set or pathway is overrepresented compared to what would be expected by chance. It is calculated as the ratio of the proportion of observed metabolites in a given pathway (or set) (*geneRatio)* to the proportion of all metabolites that are mapped to a given pathway (or set) (*BgRatio)* [(Wu et al. 2021)](https://paperpile.com/c/5gCIgH/dc7k8). The formula for the Fold Enrichment is


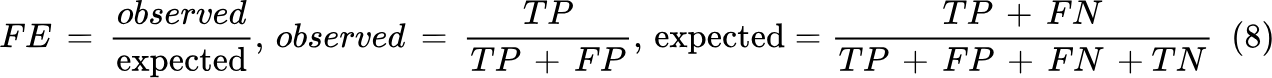


Where TP, FP, FN, TN denote true positives, false positives, false negatives and true negatives respectively.

### Isomeric ambiguity score

To quantify the isomeric/isobaric ambiguity for each ion we used Shannon’s entropy of weighted probabilities for molecular isomers associated with a particular ion. The ambiguity score quantifies how uncertain or ambiguous the assignment of a single ion is when multiple isomers are possible. The entropy is computed based on the distribution of weights assigned to its isomers. The entropy is 0 if there is only one possible isomer and for multiple isomers (N>1), the score increases as the distribution of weights becomes more uniform, indicating higher uncertainty.

To extend the ion ambiguity score to a term ambiguity score, we calculate the ratio of the intersection length between the molecules mapped to a given term and the molecular isomers associated with a particular ion, relative to the total number of isomers/isobars linked to that ion. The final score is averaged across all ions mapped to the term and scaled from 0 to 1, where a score of 1 indicates no ambiguity, and values closer to zero represent maximal ambiguity, corresponding to higher uncertainty. This score titled “*Term Ambiguity”* is scaled linearly based on the ratio of intersection with the given term, and non-linearly through an interaction between ambiguity and the intersection ratio, following an exponential decay pattern. As the intersection increases, the decay becomes less pronounced. Conversely, when the intersection is low and ion ambiguity is high, the ambiguity becomes more pronounced.


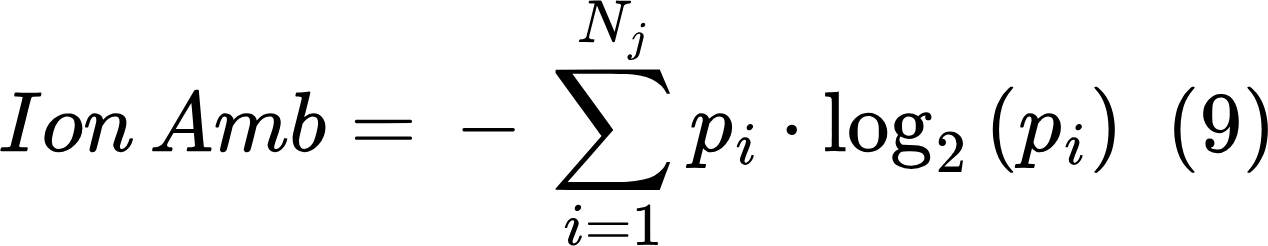


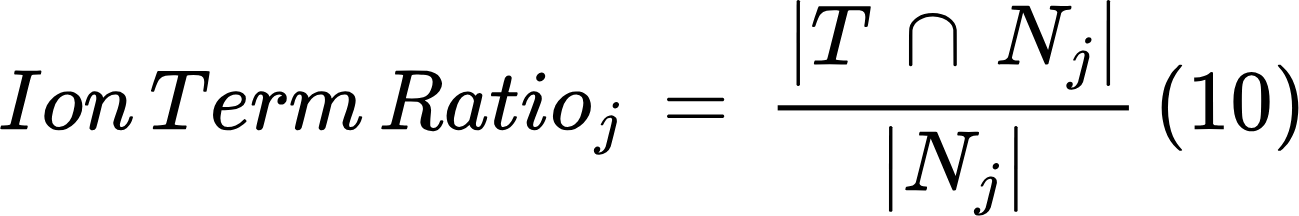


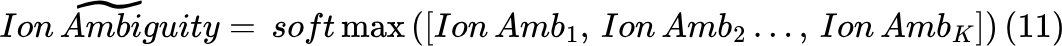


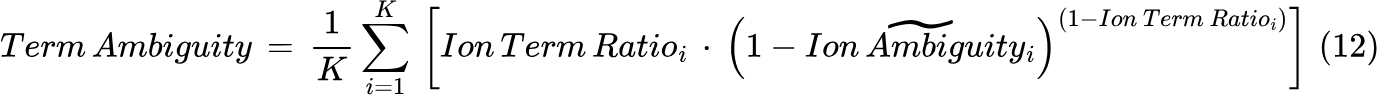


Where
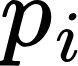
 represents the probability of each isomer/isobar,
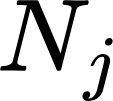
 is the total number of possible isomers/isobars for ion
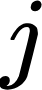
,
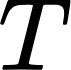
 represents the molecules associated with a given term for a given ion,
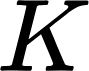
is the number of ions associated with a given term ,
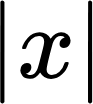
 represents the length of vector x and
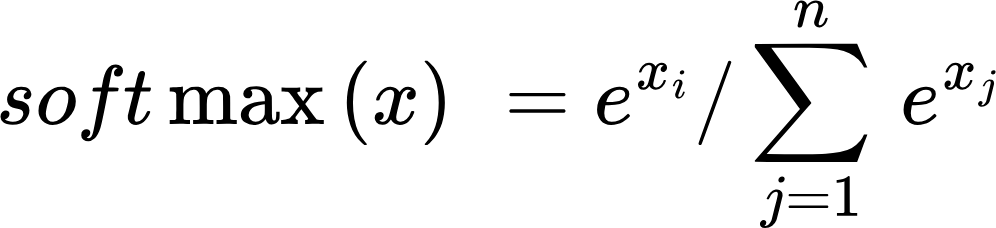


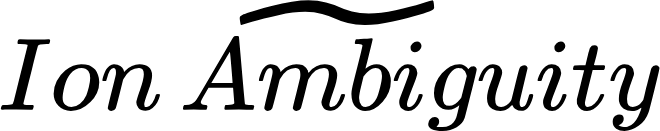
 is a vector of size
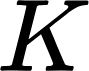
 and contains the normalized entropy for each ion associated with a given term.

When the probabilities are equally weighted, each isomer would have the same probability. If there are N isomers, the probability for each isomer is
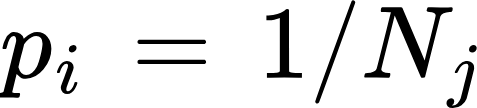
.

## **Supplementary Note 2**

The metabolite sets provided for both the METASPACE web app and S2IsoMEr were curated from either the LION ontology for lipids [(Molenaar et al. 2019)](https://paperpile.com/c/5gCIgH/AyUvH) or from RAMP-DB for metabolite- and lipid-specific classes and pathways [(Braisted et al. 2023)](https://paperpile.com/c/5gCIgH/dUdYD). The metabolite sets were constructed using the SQL database dump of RAMP-DB (v2.2.1). For metabolite classification sets, HMDB served as the source database, with only records labelled as quantified or detected being selected. The selected classification types were main, sub, or superclass categories based on the ClassyFire classification system [(Djoumbou Feunang et al. 2016)](https://paperpile.com/c/5gCIgH/taxmJ). For each class, two sets were created—one mapping terms to molecular formulas and another mapping terms to molecule names, using chemical information from the “chem_props” table in the database. For lipid classes, the same process was followed, but with LipidMaps as the source instead of HMDB. Additionally, biological pathways were mapped to lipids and metabolites using information from the “pathway” and “analytehaspathway” tables in the SQL database. For more details on the available metabolite sets, refer to the documentation for the "Load_background" function in *S2IsoMEr* (<https://alexandrovteam.github.io/S2IsoMEr/reference/Load_background.html>) .

## **Supplementary Note 3**

To visualize the enrichment results, the implemented METASPACE web app provides three main views: a table, a chart, and data filtering options. The table view displays all enrichment values associated with the current dataset, along with relevant data used for enrichment calculation. Users can export this data as a CSV file for further analysis. The chart view presents enrichment results as a bar chart, providing essential information and interactivity. By clicking on an ontology name or bar, users are redirected to the annotation page, where they can explore potential molecules involved in enrichment annotation (**Figure S3**). Lastly, the data filtering feature allows users to refine results using criteria such as ontology database, FDR, annotation database, off-sample, and p-value threshold. These filters dynamically update the table and chart views to reflect the selected parameters.

## **Supplementary Note 4**

The single-cell matrices per condition were concatenated in a single matrix and only annotations reported in METASPACE were considered. More details on samples are available in the corresponding MetaboLights record (<https://www.ebi.ac.uk/metabolights/MTBLS78>). Both ORA and MSEA analyses were conducted using the combined dataset and compared against metabolite sub-classes curated from RAMP-DB, as previously mentioned, with HMDB v4.0 [(Wishart *et al.*, 2018)](https://paperpile.com/c/k9zFE2/U96x) serving as the annotation database. Since METASPACE provides FDR-controlled annotations, we selected annotations with a 10% FDR threshold as the query set and included all reported annotations as the universe for enrichment analysis. For ORA, annotations were split into upregulated and downregulated markers based on the sign of log fold changes (LFC) between the cells of the specified conditions.

## **Supplementary Note 5**

To evaluate S2IsoMEr’s performance across different dataset sizes, bootstrap iterations, and enrichment backgrounds, we conducted a performance analysis by measuring the computational runtime of bootstrap-based ORA. We varied the number of metabolites (ranging from 8 to over 1700), the number of cells (from 2K to 20K), the number of bootstrap iterations (from 10 to 500), and the granularity of metabolite classification, which includes different number of terms —from 25 at the superclass level to 600 at the subclass level. The results indicate that runtime scales almost linearly with the number of bootstrap iterations and is influenced by the enrichment background, while remaining robust to variations in the number of cells and metabolites (**Figure S7**).

## **Supplementary Figures**

##
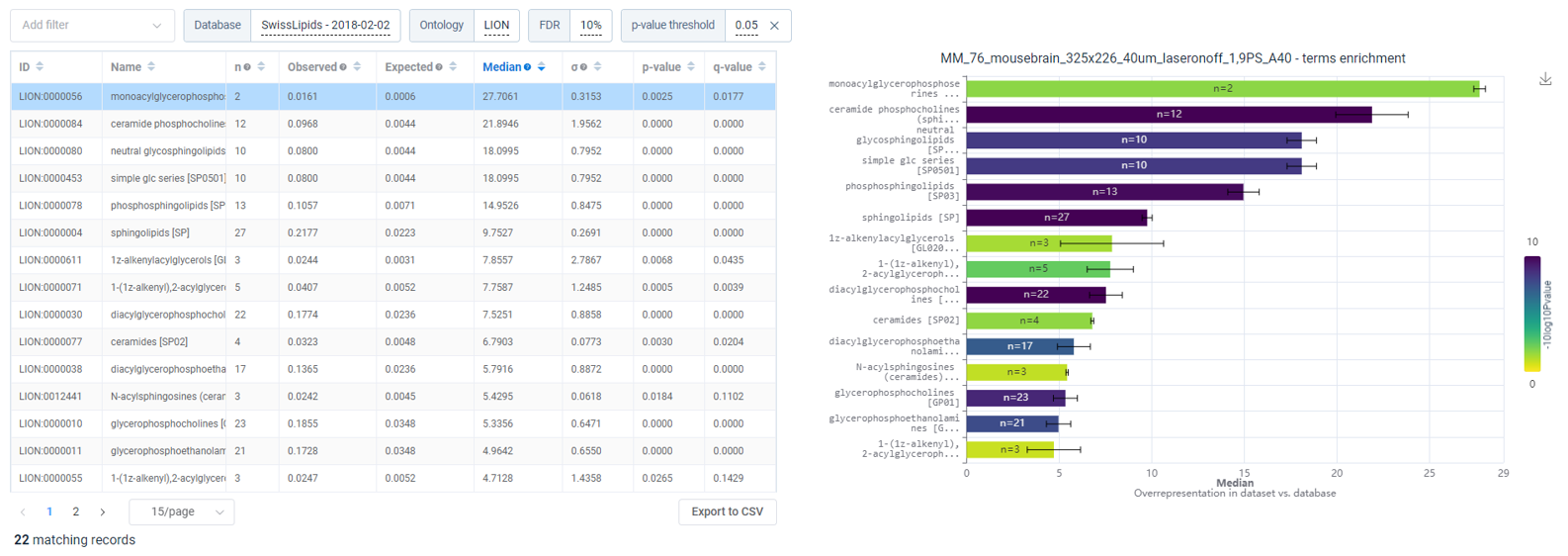


*Figure S1: METASPACE Enrichment web app*

Screenshot from METASPACE web app enrichment results on a brain datasets (<https://metaspace2020.eu/dataset/2022-05-31_10h46m34s>) using FDR 10%, a p-value threshold of 0.05, LION ontology as metabolite set and SwissLipids as annotation database.

##
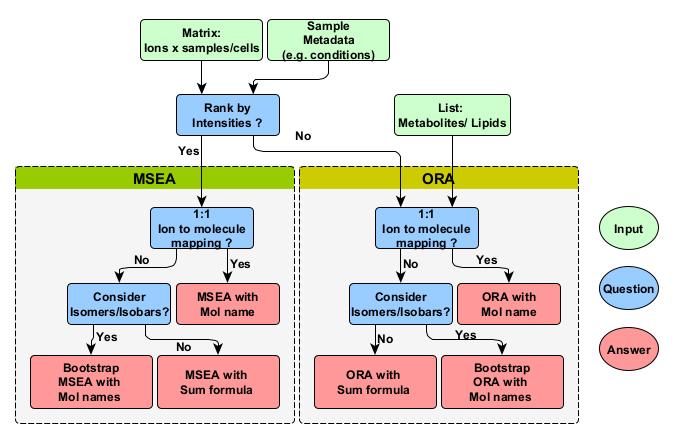


*Figure S2: Enrichment Types Decision Tree*

Flowchart to help the user decide on which enrichment type would be appropriate based on the provided input data. Nodes coloured in green, blue and red represent input, questions and answers, respectively.


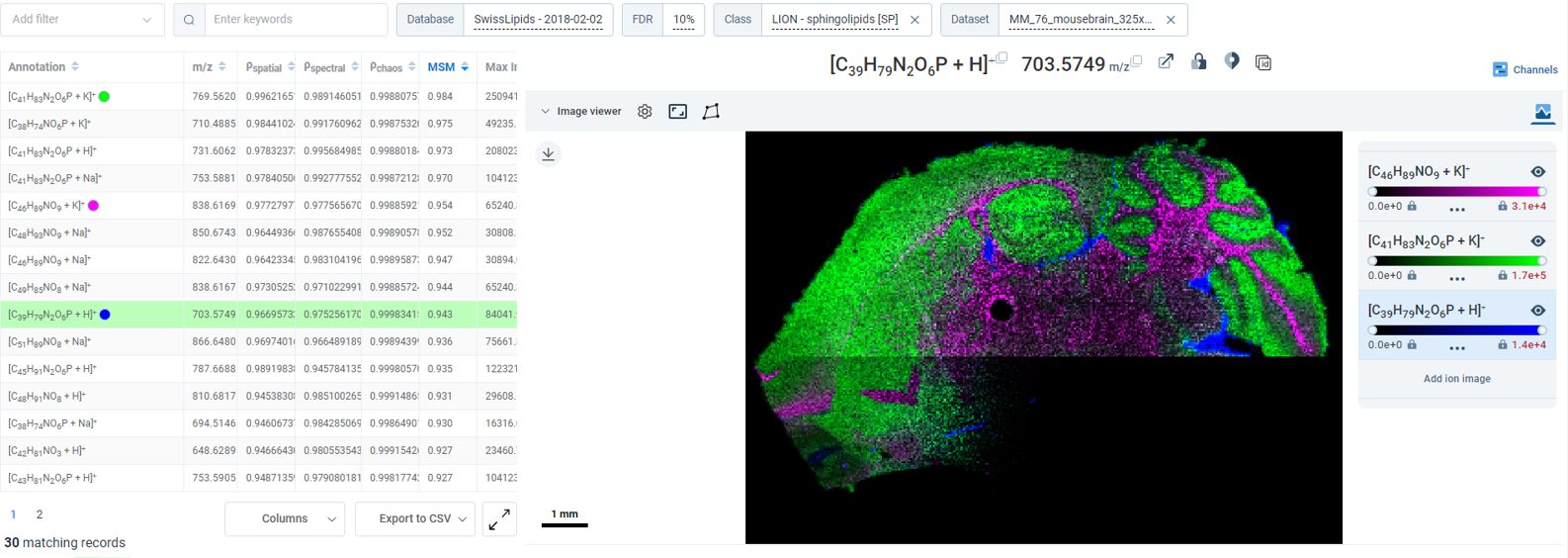


*Figure S3: Sphingolipids annotations in mouse brain*

Screenshot from annotation page on METASPACE for brain dataset (<https://metaspace2020.eu/dataset/2022-05-31_10h46m34s>) after clicking on sphingolipids bar in *Figure S1.*


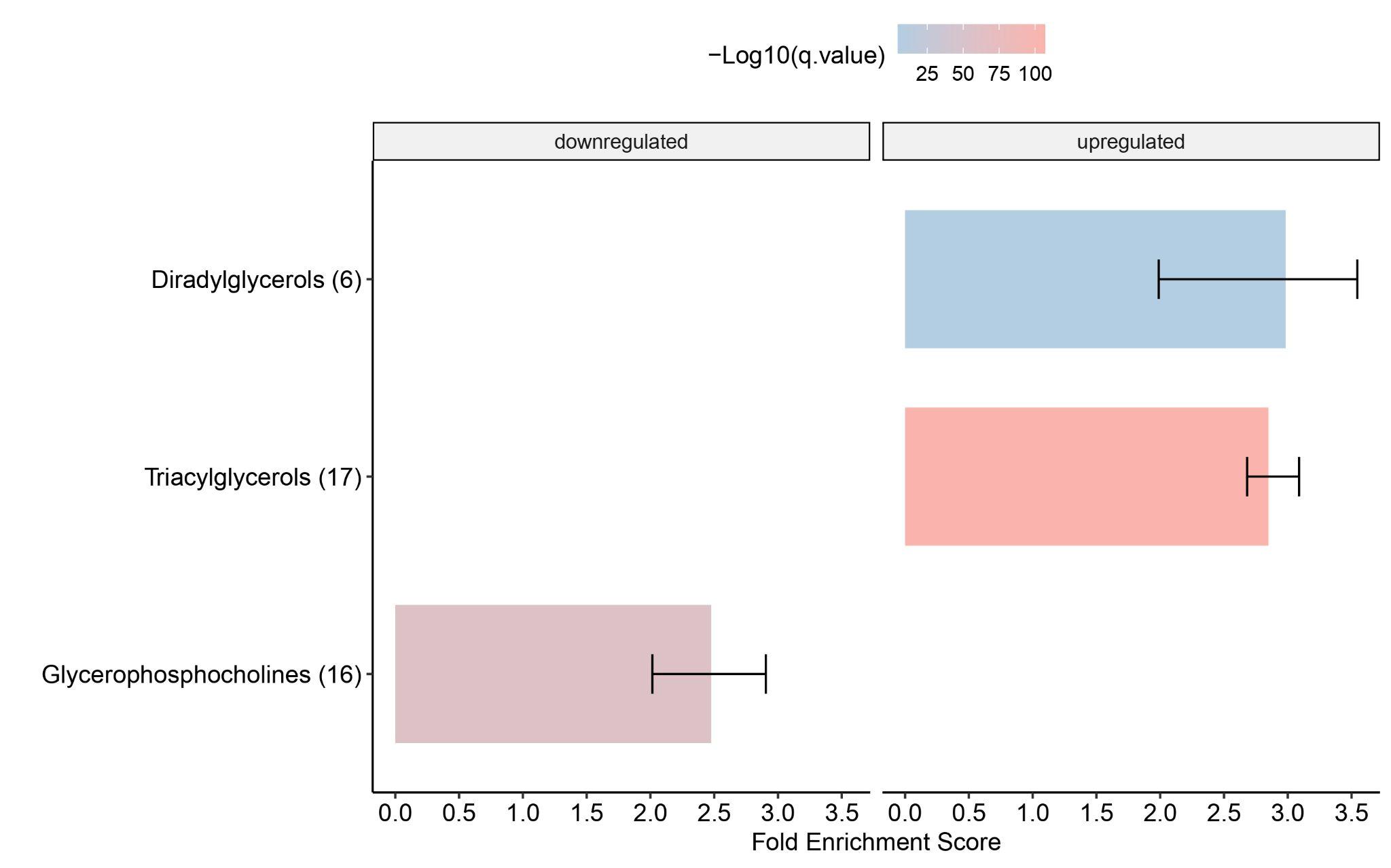


*Figure S4: ORA results for SpaceM NASH dataset*

Barplot showing results of bootstrapping-based ORA on a single-cell dataset based on a NASH model from SpaceM (<https://www.ebi.ac.uk/metabolights/MTBLS78>). Enriched terms are plotted on the y-axis and fold enrichment score on the x-axis. Bar colour corresponds to -log10 (q-value) and error bars represent min and max enrichment scores across bootstrap iterations. Size of term/query overlap (i.e. number of molecules) are displayed in parentheses next to enriched terms.


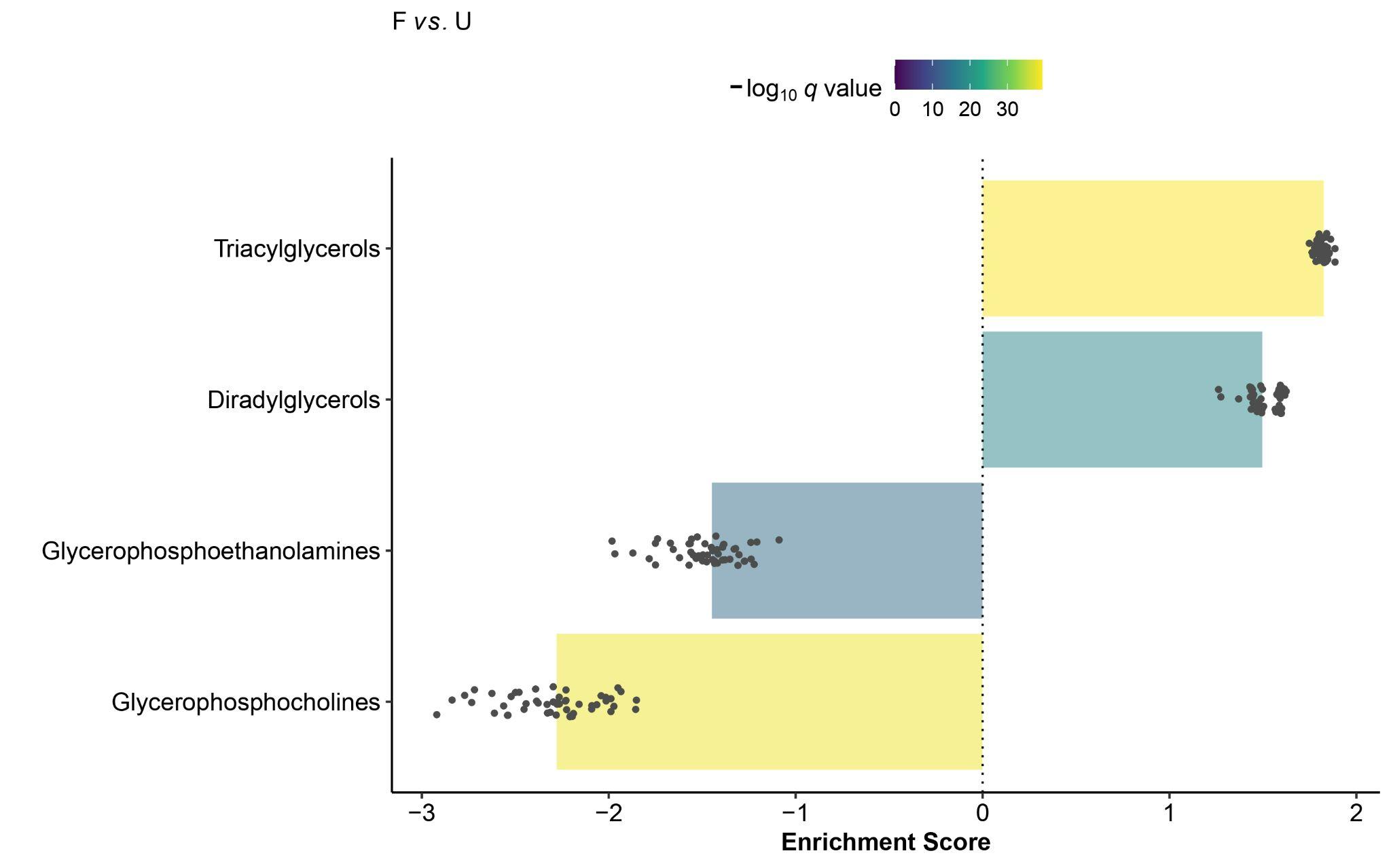


*Figure S5: MSEA results for SpaceM NASH dataset*

Barplot showing results of bootstrapping-based MSEA on a single-cell dataset based on a NASH model from SpaceM (<https://www.ebi.ac.uk/metabolights/MTBLS78>). Enriched terms are plotted on the y-axis and normalized enrichmed score (NES) on the x-axis. Bar colour corresponds to -log10 (q-value) and jittered points represent NES scores for each bootstrap iteration.


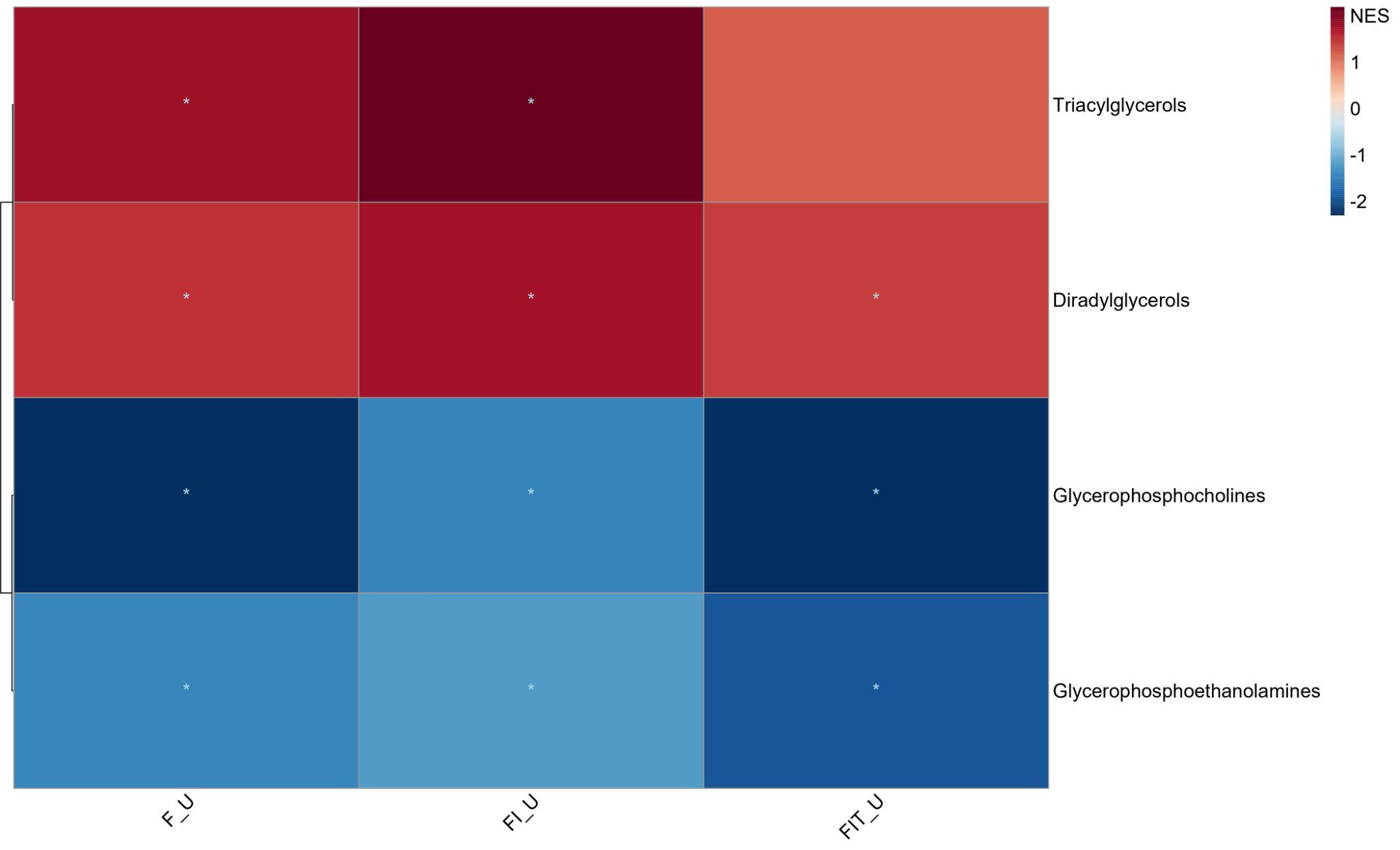


*Figure S6: MSEA Multi-condition results*

Heatmap showing MSEA results of bootstrapping-based MSEA on a single-cell dataset based on a NASH model from SpaceM (<https://www.ebi.ac.uk/metabolights/MTBLS78>). Rows represent the enriched terms and columns representing pairwise conditions as “test_reference”. Color scale corresponds to normalized enrichment score (NES).


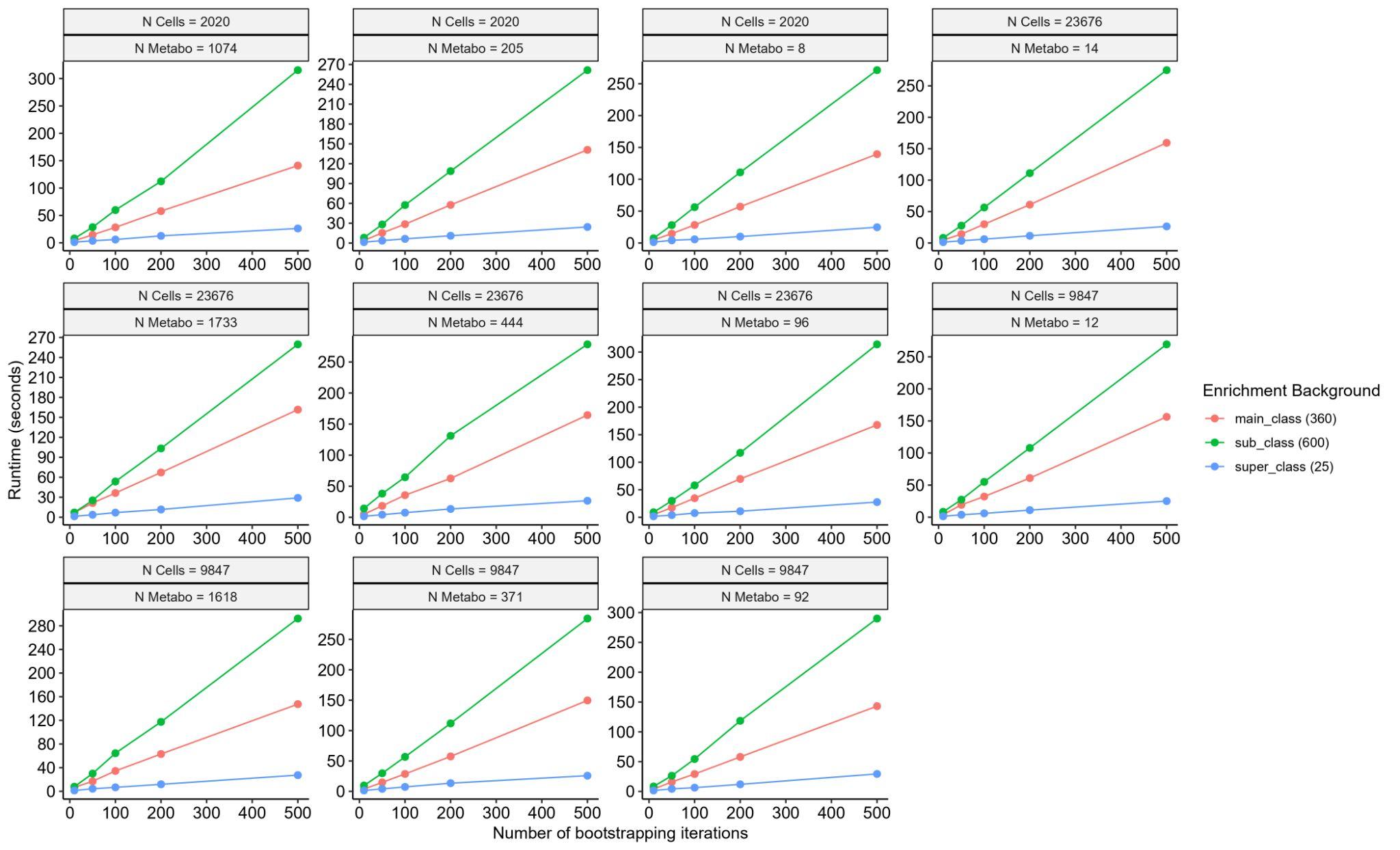


*Figure S7: Performance analysis results*

Line plots showing computational runtime in seconds (y-axis) as a function of the number of bootstrap iterations (x-axis). Each panel represents a different dataset size, specified by the number of cells and metabolites in the panel title. Lines are color-coded and grouped by enrichment background (superclass, main class, or subclass), with the number of terms in each class indicated in parentheses.


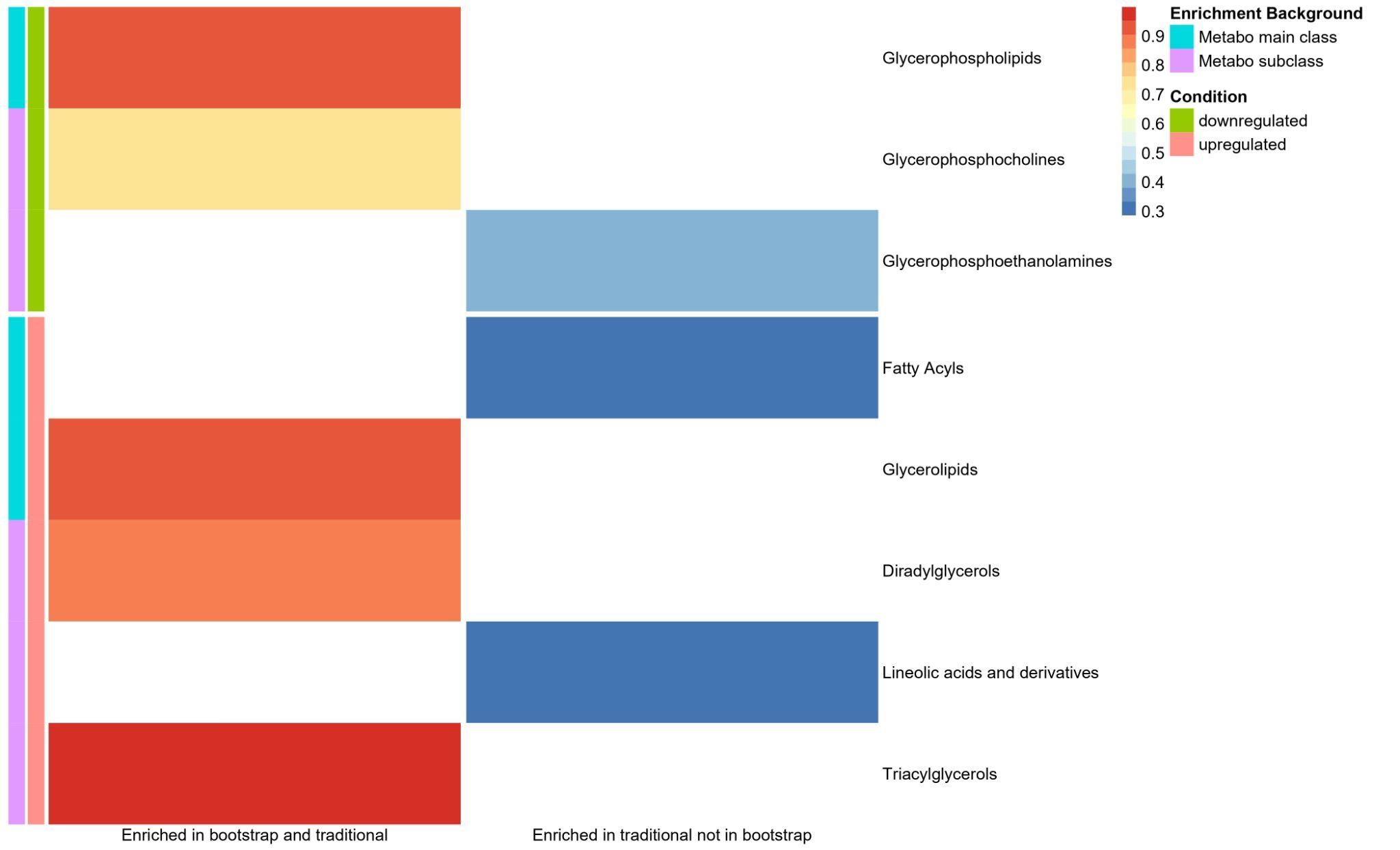


*Figure S8: Traditional vs bootstrap-based ORA*

Heatmap displaying significantly enriched terms from traditional ORA, grouped by metabolic class type and condition as row annotation bars. Rows display the enriched terms, while columns indicate whether the terms are significantly enriched in the bootstrap-based approach or not. The color scale represents the term ambiguity score (Eq 12).

## References

[Badia-I-Mompel, Pau, Jesús Vélez Santiago, Jana Braunger, Celina Geiss, Daniel Dimitrov, Sophia Müller-Dott, Petr Taus, et al. 2022. “decoupleR: Ensemble of Computational Methods to Infer Biological Activities from Omics Data.” *Bioinformatics Advances* 2 (1): vbac016.](http://paperpile.com/b/5gCIgH/BoLmi)

[Braisted, John, Andrew Patt, Cole Tindall, Timothy Sheils, Jorge Neyra, Kyle Spencer, Tara Eicher, and Ewy A. Mathé. 2023. “RaMP-DB 2.0: A Renovated Knowledgebase for Deriving Biological and Chemical Insight from Metabolites, Proteins, and Genes.” *Bioinformatics*  39 (1). https://doi.org/](http://paperpile.com/b/5gCIgH/dUdYD)[10.1093/bioinformatics/btac726](http://dx.doi.org/10.1093/bioinformatics/btac726)[.](http://paperpile.com/b/5gCIgH/dUdYD)

[Djoumbou Feunang, Yannick, Roman Eisner, Craig Knox, Leonid Chepelev, Janna Hastings, Gareth Owen, Eoin Fahy, et al. 2016. “ClassyFire: Automated Chemical Classification with a Comprehensive, Computable Taxonomy.” *Journal of Cheminformatics* 8 (November):61.](http://paperpile.com/b/5gCIgH/taxmJ)

[Korotkevich, Gennady, Vladimir Sukhov, Nikolay Budin, Boris Shpak, Maxim N. Artyomov, and Alexey Sergushichev. 2021. “Fast Gene Set Enrichment Analysis.” *bioRxiv*. https://doi.org/](http://paperpile.com/b/5gCIgH/AXznV)[10.1101/060012](http://dx.doi.org/10.1101/060012)[.](http://paperpile.com/b/5gCIgH/AXznV)

[Molenaar, Martijn R., Aike Jeucken, Tsjerk A. Wassenaar, Chris H. A. van de Lest, Jos F. Brouwers, and J. Bernd Helms. 2019. “LION/web: A Web-Based Ontology Enrichment Tool for Lipidomic Data Analysis.” *GigaScience* 8 (6). https://doi.org/](http://paperpile.com/b/5gCIgH/AyUvH)[10.1093/gigascience/giz061](http://dx.doi.org/10.1093/gigascience/giz061)[.](http://paperpile.com/b/5gCIgH/AyUvH)

[Murakami, Hidetoshi. 2012. “Modified Baumgartner Statistics for the Two-Sample and Multisample Problems: A Numerical Comparison.” *Journal of Statistical Computation and Simulation* 82 (5): 711–28.](http://paperpile.com/b/5gCIgH/fJ8jX)

[Wu, Tianzhi, Erqiang Hu, Shuangbin Xu, Meijun Chen, Pingfan Guo, Zehan Dai, Tingze Feng, et al. 2021. “clusterProfiler 4.0: A Universal Enrichment Tool for Interpreting Omics Data.” *Innovation (Cambridge (Mass.))* 2 (3): 100141.](http://paperpile.com/b/5gCIgH/dc7k8)

[Zyla, Joanna, Michal Marczyk, January Weiner, and Joanna Polanska. 2017. “Ranking Metrics in Gene Set Enrichment Analysis: Do They Matter?” *BMC Bioinformatics* 18 (1): 256.](http://paperpile.com/b/5gCIgH/qWz7W)
