# Supplementary figures and images for: Enrichment analysis for spatial and single-cell metabolomics accounting for molecular ambiguity
Source: Bioinform Adv. 2025 May 21;5(1):vbaf100. doi: 10.1093/bioadv/vbaf100 (PMC12158160; doi:10.1093/bioadv/vbaf100)

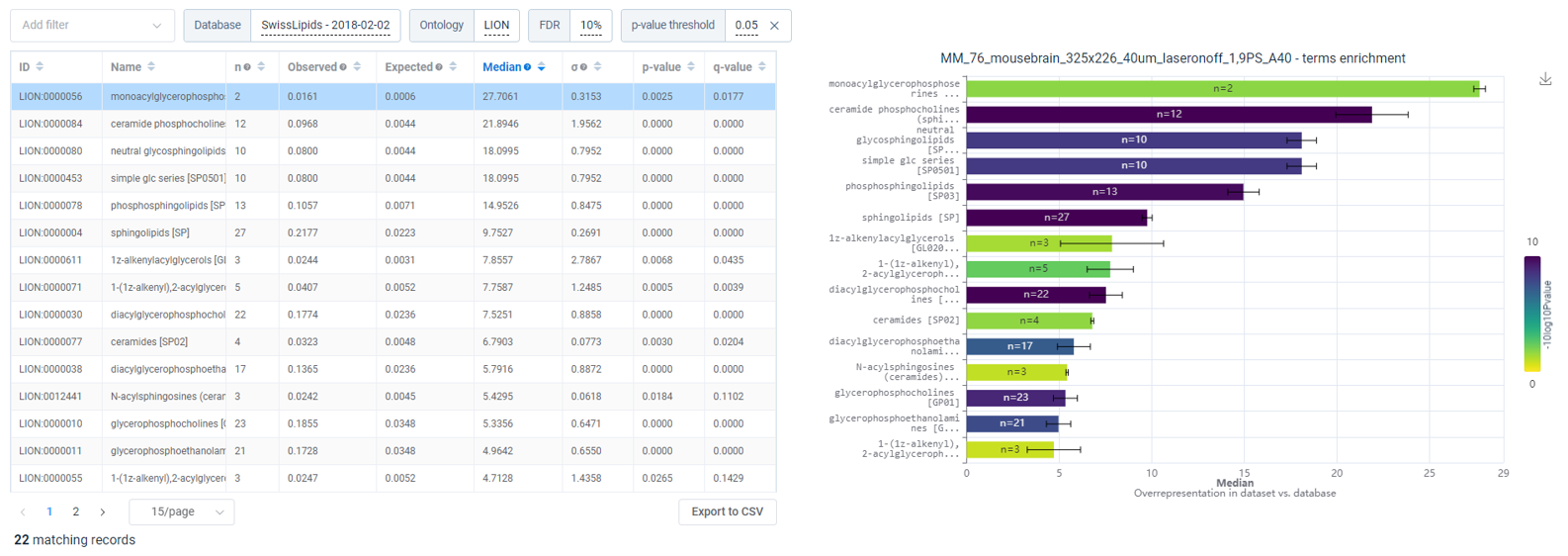

Supplement: vbaf100_Supplementary_Data [file vbaf100_supplementary_data.zip › Figure S1.tiff]

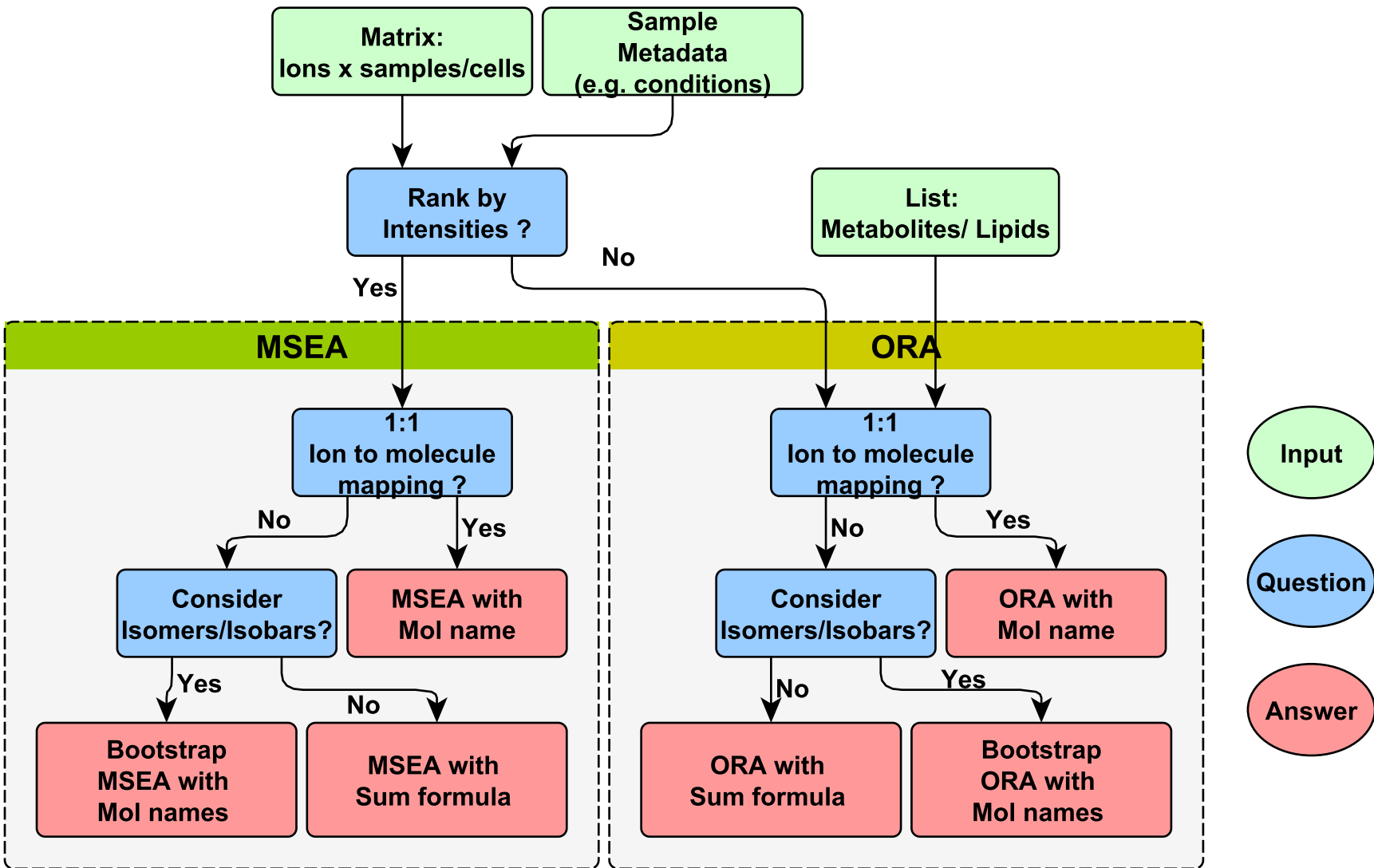

Supplement: vbaf100_Supplementary_Data [file vbaf100_supplementary_data.zip › Figure S2.pdf]

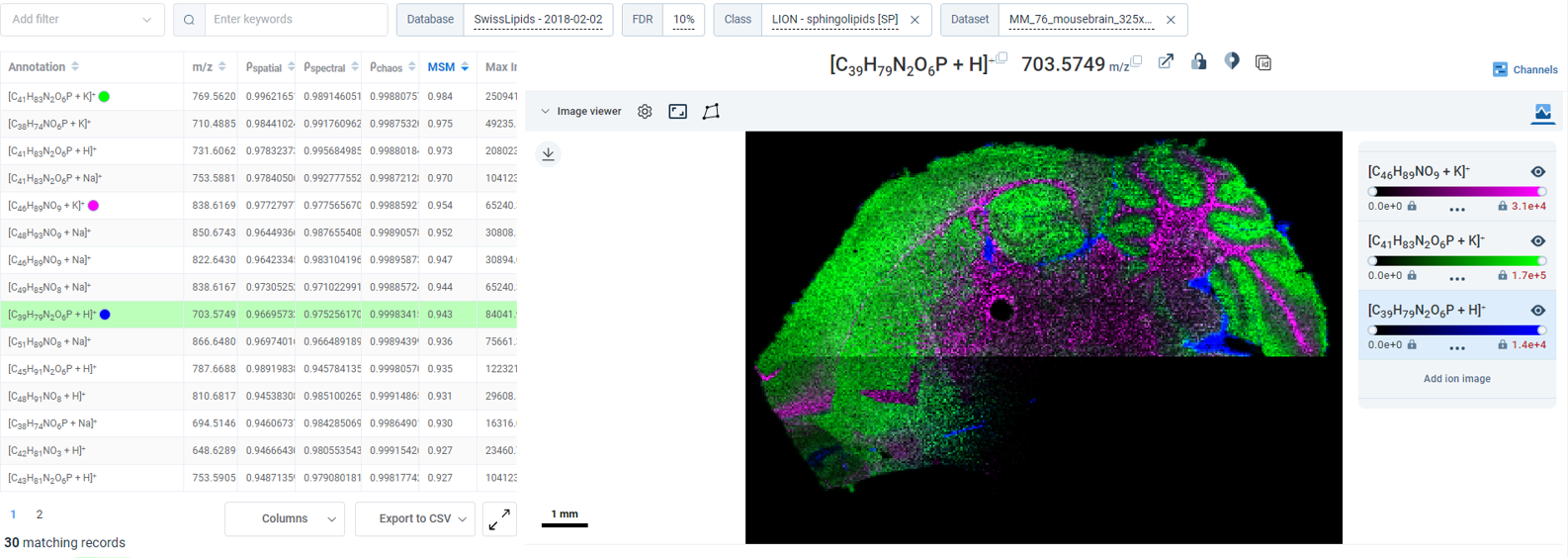

Supplement: vbaf100_Supplementary_Data [file vbaf100_supplementary_data.zip › Figure S3.tiff]

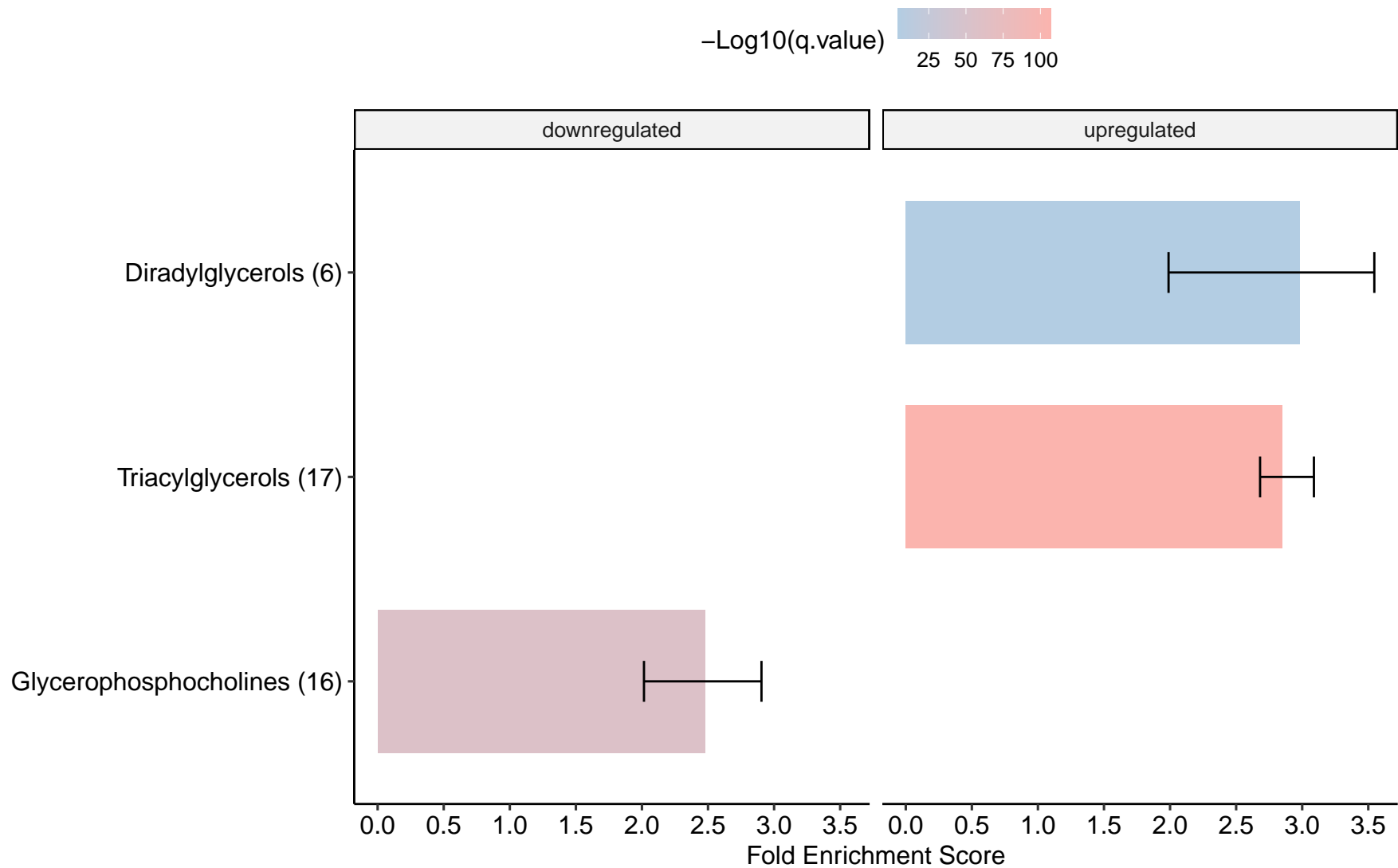

Supplement: vbaf100_Supplementary_Data [file vbaf100_supplementary_data.zip › Figure S4.pdf]

F vs. U

$-\log_{10} q \text{ value}$

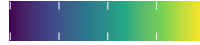

0 10 20 30

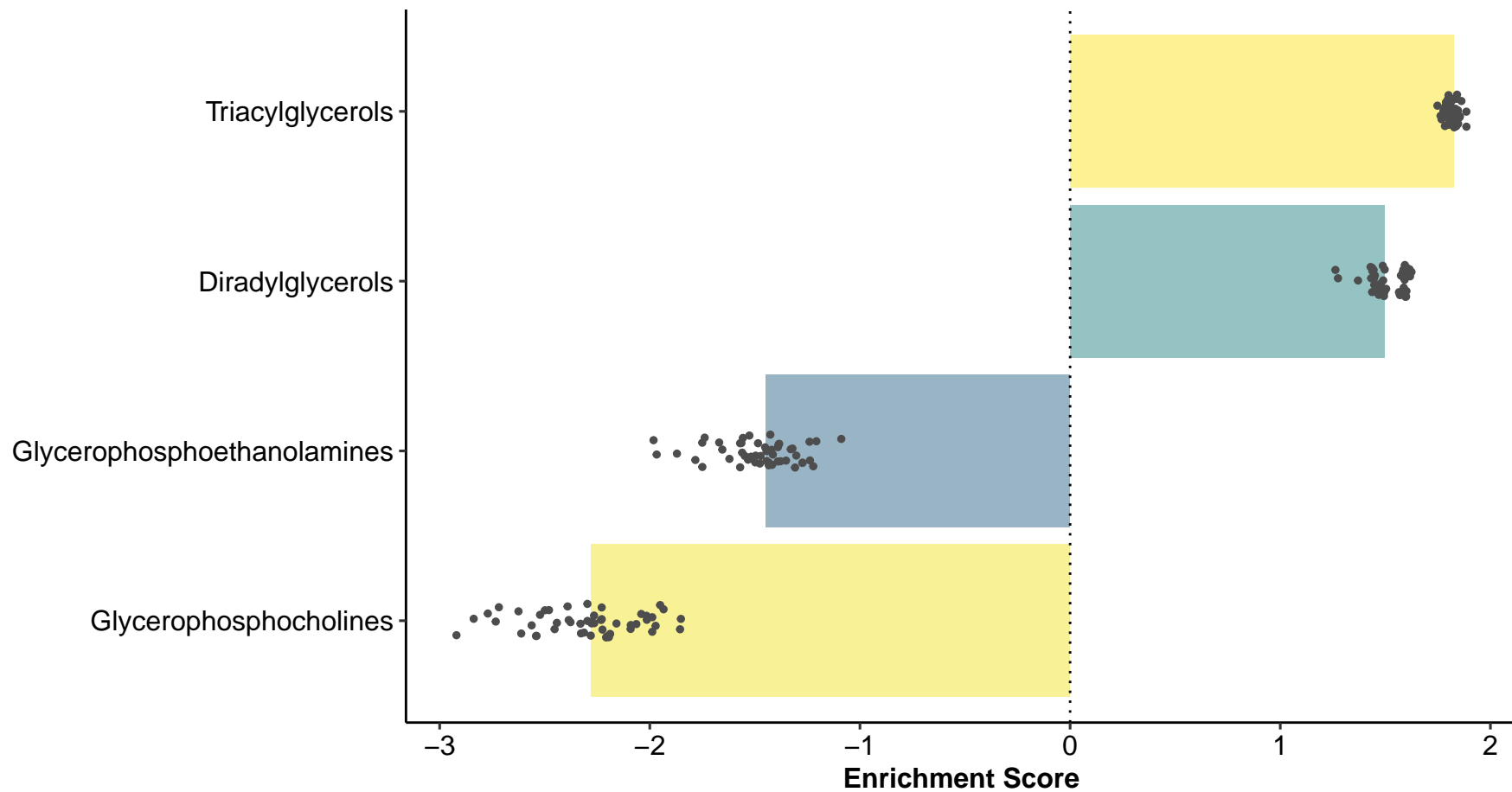

Supplement: vbaf100_Supplementary_Data [file vbaf100_supplementary_data.zip › Figure S5.pdf]

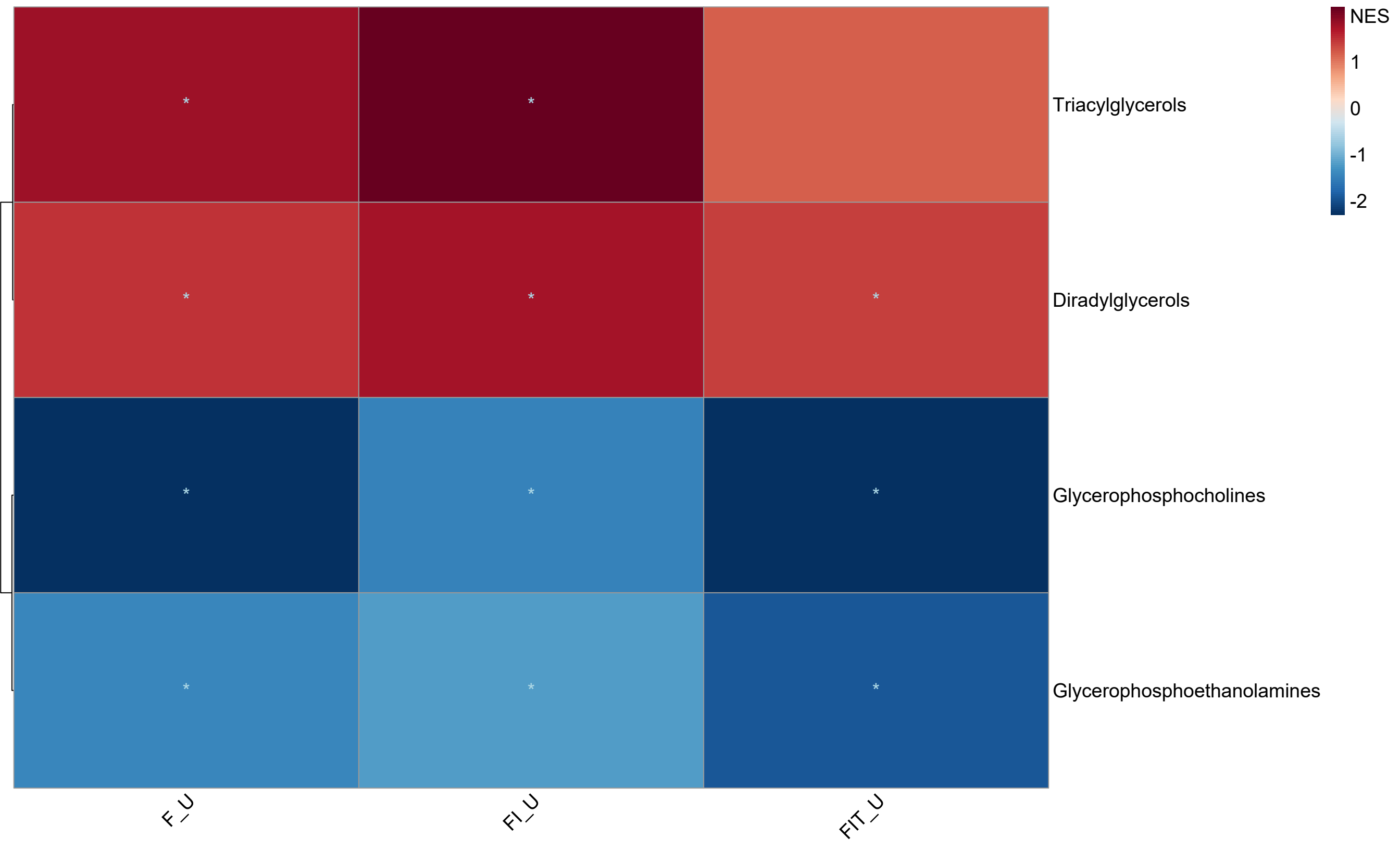

Supplement: vbaf100_Supplementary_Data [file vbaf100_supplementary_data.zip › Figure S6.pdf]

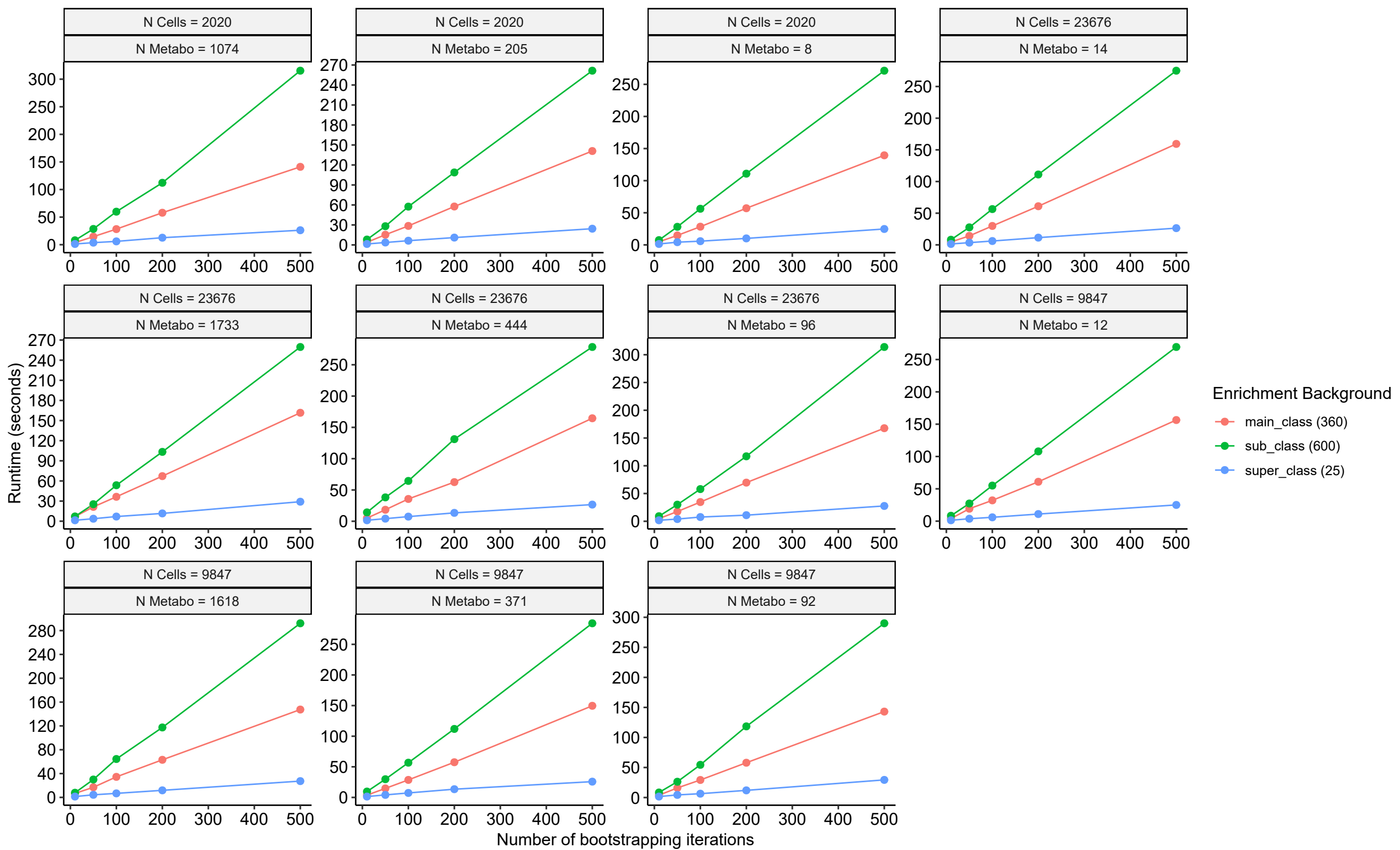

Supplement: vbaf100_Supplementary_Data [file vbaf100_supplementary_data.zip › Figure S7.pdf]

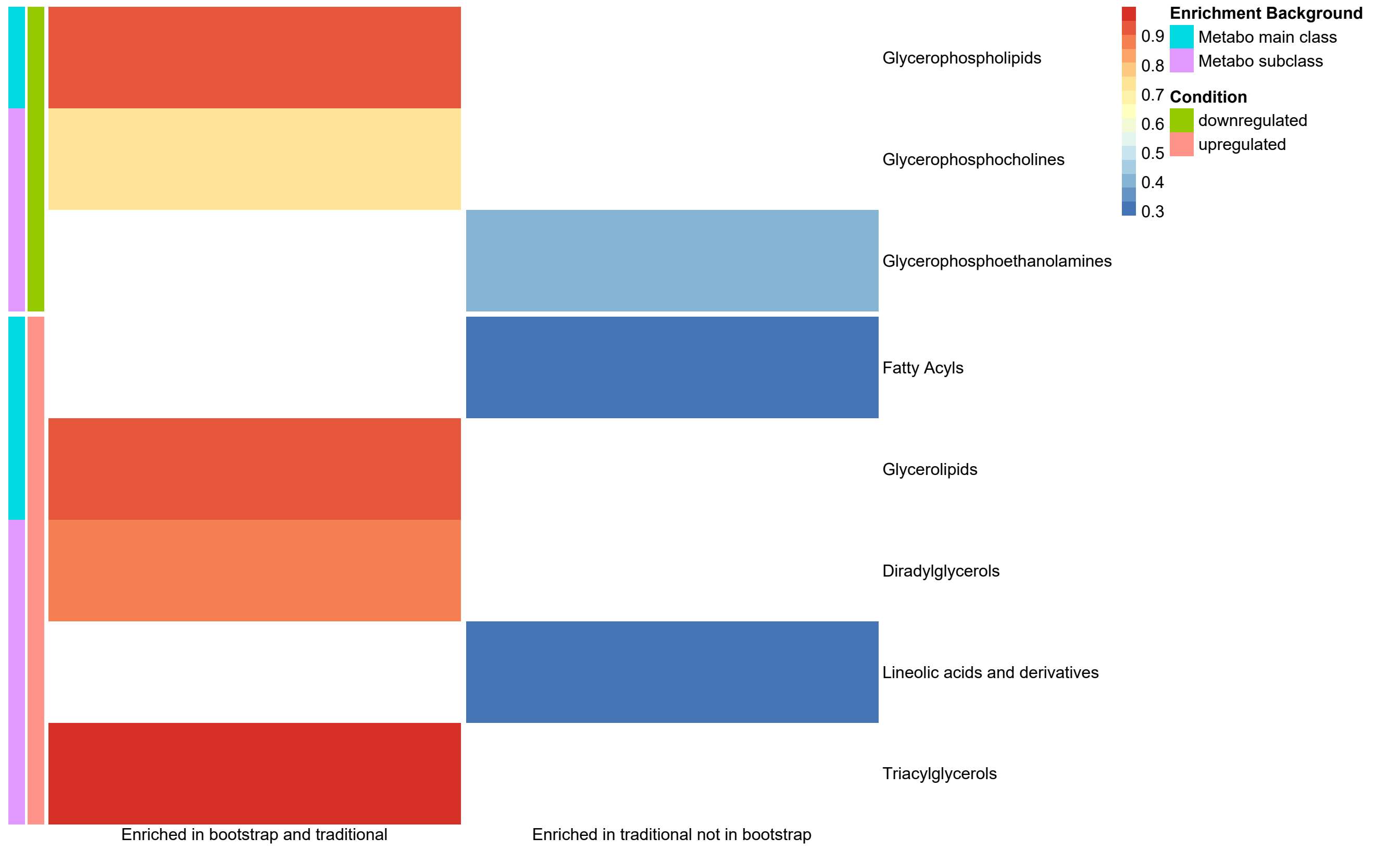

Supplement: vbaf100_Supplementary_Data [file vbaf100_supplementary_data.zip › Figure S8.pdf]
